# Supplementary material for: Identifying repeat domains in large genomes
Source: Genome Biol. 2006 Jan 31;7(1):R7. doi: 10.1186/gb-2006-7-1-r7 (PMC1431705; doi:10.1186/gb-2006-7-1-r7)
Supplement: Additional File 1 — A zipped file of browsable HTML files with a complete list of the connected components in the repeat domain graph of human Repbase. [file gb-2006-7-1-r7-S1.gz › html/subgraphs/6841.pdf]

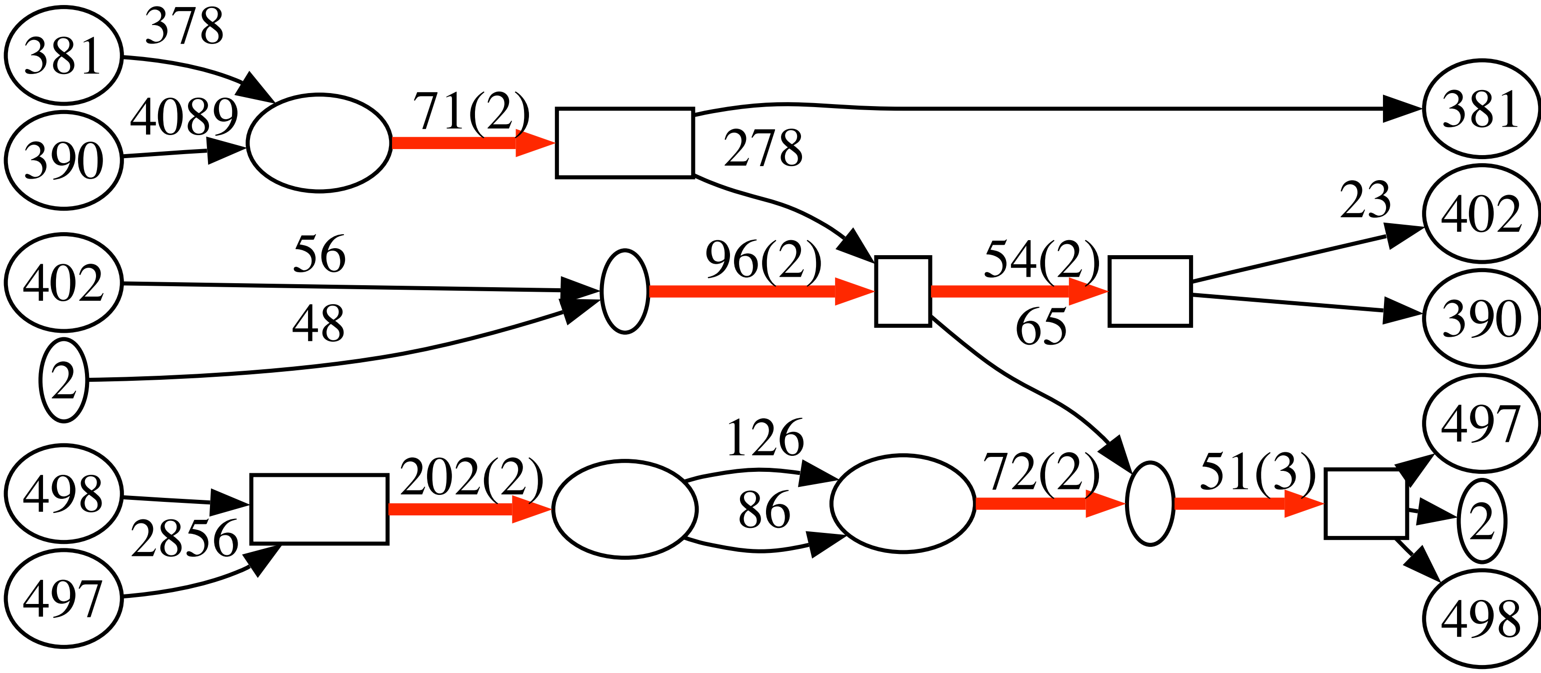

381
378
390
4089
71(2)
278
381
23
402
402
56
96(2)
54(2)
48
65
390
2
498
126
202(2)
72(2)
51(3)
497
86
2
2856
497
498
